# Regulatory Noncoding Small RNAs Are Diverse and Abundant in an Extremophilic Microbial Community

**DOI:** 10.1128/mSystems.00584-19

**Published:** 2020-02-04

**Authors:** Diego R. Gelsinger, Gherman Uritskiy, Rahul Reddy, Adam Munn, Katie Farney, Jocelyne DiRuggiero

**Affiliations:** a Department of Biology, The Johns Hopkins University, Baltimore, Maryland, USA; California State University, Northridge

**Keywords:** RNA, extremophiles, gene regulation, metagenomics, metatranscriptomics, microbial communities, microbiome, noncoding

## Abstract

Regulatory small RNAs (sRNAs) play large-scale and essential roles in many cellular processes across all domains of life. Microbial sRNAs have been extensively studied in model organisms, but very little is known about the dynamics of sRNA synthesis and their roles in the natural environment. In this study, we discovered hundreds of intergenic (itsRNAs) and antisense (asRNAs) sRNAs expressed in an extremophilic microbial community inhabiting halite nodules (salt rocks) in the Atacama Desert. For this, we built SnapT, a new sRNA annotation pipeline that can be applied to any microbial community. We found asRNAs with expression levels negatively correlated with that of their overlapping putative target and itsRNAs that were conserved and significantly differentially expressed between 2 sampling time points. We demonstrated that we could perform target prediction and correlate expression levels between sRNAs and predicted target mRNAs at the community level. Functions of putative mRNA targets reflected the environmental challenges members of the halite communities were subjected to, including osmotic adjustments to a major rain event and competition for nutrients.

**IMPORTANCE** Microorganisms in the natural world are found in communities, communicating and interacting with each other; therefore, it is essential that microbial regulatory mechanisms, such as gene regulation affected by small RNAs (sRNAs), be investigated at the community level. This work demonstrates that metatranscriptomic field experiments can link environmental variation with changes in RNA pools and have the potential to provide new insights into environmental sensing and responses in natural microbial communities through noncoding RNA-mediated gene regulation.

## Abstract (precis)

Microorganisms in the natural world are found in communities, communicating and interacting with each other; therefore, it is essential that microbial regulatory mechanisms, such as gene regulation affected by small RNAs (sRNAs), be investigated at the community level. This work demonstrates that metatranscriptomic field experiments can link environmental variation with changes in RNA pools and have the potential to provide new insights into environmental sensing and responses in natural microbial communities through noncoding RNA-mediated gene regulation.

## INTRODUCTION

Noncoding RNAs (ncRNAs) are untranslated short transcripts that are found in the three domains of life, where they play essential roles in many cellular processes (1, 2). In prokaryotes, a subset of these ncRNAs, thereby called small RNAs (sRNAs), is specifically involved in gene regulation through RNA-RNA mediated interactions, modulating core metabolic functions and stress-related responses (3). These sRNAs range from 50 to 500 nucleotides in size and can be of two types. *trans*-encoded sRNAs, also called intergenic sRNAs (itsRNAs), bind their mRNA targets via imperfect base pairing and can target multiple genes, including key transcription factors and regulators (4). itsRNAs can activate or inhibit translation initiation by interacting with the ribosome binding site (RBS) and/or modulating mRNA stability (4). In contrast, *cis*-encoded antisense RNAs (asRNAs) are transcribed on the DNA strand opposite their target gene and thus can act via extensive base pairing; they have been found to repress transposons and toxic protein synthesis (4).

The functional roles of microbial sRNAs have been extensively studied in a few model organisms, and very little is known about the dynamics of sRNA synthesis in natural environments and the roles of these short transcripts at the community level (1, 5). To our knowledge, only a few studies have reported the discovery of sRNAs in natural microbial communities (6–9), and there is no publicly available bioinformatic tool for sRNA discovery in single-species isolates and in the metagenomic context (1). This paucity of knowledge suggests that an abundance of sRNAs remain to be discovered with potentially essential roles in stress response (10), interspecies communication, and/or cross-species RNA interference (11–13). This might be relevant to extreme environments where microbial communities are specifically adapted to a narrow set of environmental conditions, i.e., high salt and low pH, and are particularly sensitive to perturbations (14).

In hyperarid deserts, microbial communities find refuge inside rocks as a survival strategy against the extreme conditions of their environment (15). Such a community inhabits halite (salt) nodules in Salars of the Atacama Desert, Chile, which is one of the oldest and driest deserts on Earth (16, 17). The halite endolithic (within rock) community harbors mostly members of the *Archaea* (*Halobacteria*), unique *Cyanobacteria*, diverse heterotrophic bacteria, and a novel type of algae (16, 17), all of which were shown to be transcriptionally active (18). The main source of liquid water for this community is from salt deliquescence (19), and it is sustained by CO_2_ fixed via photosynthesis (16, 20). While previous studies have demonstrated the role of sRNAs in the stress response of one of the members of this community, the halophilic archaeon Haloferax volcanii (21, 22), there is no information on any of the other members.

Here, we used a combination of genome-resolved metagenomics and metatranscriptomics to investigate the role of sRNAs in the adaptive response of microorganisms inhabiting halite nodules. We developed an analytical pipeline, SnapT, built on our previous work on sRNAs from the archaeon H. volcanii (21), to enable the discovery of sRNAs at the community level. Using strand-specific metatranscriptomics, we found hundreds of sRNAs (both itsRNAs and asRNAs) from multiple trophic levels in the halite community, including conserved sRNAs, validating our experimental approach. Previous studies were limited to either intergenic or antisense sRNAs, never both; analysis of both types of sRNAs in our study allowed for the most comprehensive view of the sRNA regulatory landscape in a microbial community (6–9). A number of itsRNAs were significantly differentially regulated between 2 sampling time points, providing validation that sRNAs can be modulated in the natural environment. For a subset of these, we were able to perform structure and target prediction of conserved sRNAs to decipher their potential regulatory roles, a first at the metatranscriptomic level. Coupling metagenomics and metatranscriptomics with SnapT allows for the potential to uncover the complex regulatory networks that govern the state of a microbial community.

## RESULTS

### Landscape of predicted sRNAs in the halite community and validation.

We discovered hundreds of ncRNAs in an extremophilic community inhabiting halite nodules (salt rocks) in the Atacama Desert by using SnapT (https://github.com/ursky/SnapT), a bioinformatic tool for sRNA discovery (Table 1; see also Data Set S1 in the supplemental material). We used metatranscriptomics data from multiple replicate samples collected in the field in 2016 and 2017 (21 and 24 replicates for 2016 and 2017, respectively) (see Table S1). Using SnapT, we aligned reads from stranded RNA sequencing (RNA-seq) libraries to our reference coassembled metagenome from a previous study (14) (See Fig. S1). The assembled transcripts were then intersected with the metagenome annotation as well as open reading frames to select for either novel transcripts on the opposite strand of coding transcripts (asRNAs) or novel transcripts that fell into intergenic regions (itsRNAs). Putative ncRNA transcripts were then further enriched using thresholds at 5× and 10× assembly coverage to identify intergenic and antisense ncRNAs, respectively. (Table 1; see Fig. S2A). The size of these ncRNAs was then filtered from 50 to 500 nucleotides to produce a final set of noncoding sRNAs. The size distribution of these sRNAs was primarily between 50 and 200 nt for itsRNAs and >200 nt for asRNAs. (Fig. S2B and C).

**TABLE 1:** Summary of ncRNAs discovered in halite community

RNA type	No. (%)^*a*^	% in *Archaea*	% in *Bacteria*
Total ncRNA	1,538 (100)	54	46
Rfam ncRNA	79 (5)	73	27
Conserved sRNA^*b*^	155 (10)	60	40
Antisense sRNA	925 (60)	40	60
Intergenic sRNA	613 (40)	75	25

a Percent from total ncRNAs.

b Conserved other than Rfam ncRNAs.

10.1128/mSystems.00584-19.1 FIG S1 Flow chart for SnapT methodology. Download FIG S1, TIF file, 2.5 MB. Copyright © 2020 Gelsinger et al. 2020 Gelsinger et al. This content is distributed under the terms of the Creative Commons Attribution 4.0 International license.

10.1128/mSystems.00584-19.2 FIG S2 Properties of sRNAs. (A) Thresholding for itsRNAs and asRNAs. Ranked total expression (transcripts per million) of annotated antisense and intergenic small ncRNAs. The figure shows the linear relationship between the sRNA expression in TPM and the number of sRNAs and how it decays. A threshold at 5× and 10× coverage was applied to itsRNAs and asRNAs, respectively. Length distributions of itsRNAs (B) and asRNAs (C). (D) Rfam conserved sRNAs identified the halite community. (E) asRNA overlap distribution with their putative mRNAs. Download FIG S2, TIF file, 1.3 MB. Copyright © 2020 Gelsinger et al. 2020 Gelsinger et al. This content is distributed under the terms of the Creative Commons Attribution 4.0 International license.

10.1128/mSystems.00584-19.8 DATA SET S1 sRNA features with expression levels, conserved sRNAs, Rfam hits, asRNA gene pairs, significantly differentially expressed sRNAs, and IntaRNA interactions for itsRNAs. Download Data Set S1, XLSX file, 10.8 MB. Copyright © 2020 Gelsinger et al. 2020 Gelsinger et al. This content is distributed under the terms of the Creative Commons Attribution 4.0 International license.

10.1128/mSystems.00584-19.9 TABLE S1 Samples collected and sequenced libraries. Download Table S1, PDF file, 0.05 MB. Copyright © 2020 Gelsinger et al. 2020 Gelsinger et al. This content is distributed under the terms of the Creative Commons Attribution 4.0 International license.

**FIG 1:**
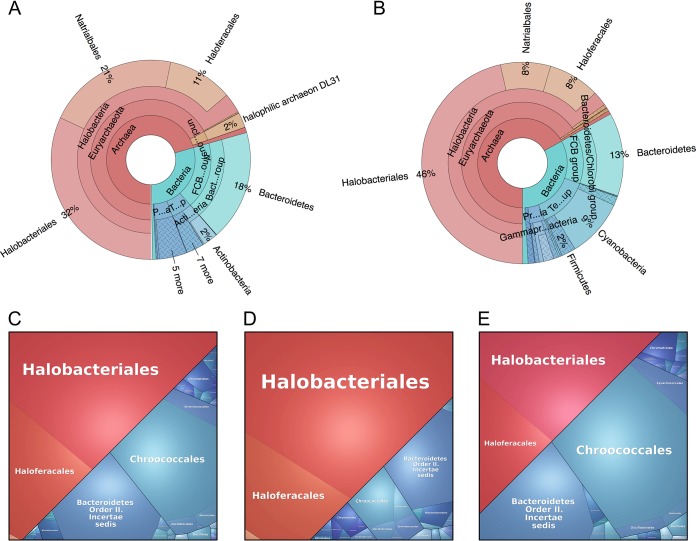
Taxonomic distribution. Krona graphs of the halite metagenome based of DNA sequence reads (A) and the halite metatranscriptome based on RNA sequence reads (B). Voronoi plots of total sRNAs (C), itsRNAs (D), and asRNAs (E) discovered in the halite community. Partitions of the Voronoi plots correspond to relative abundances of the indicated taxa.

The halite ncRNAs were taxonomically assigned to diverse members of the community; their distributions between *Archaea* (54%) and *Bacteria* (46%) (Table 1) were similar to that of the total metatranscriptomic reads for the community (Fig. 1B and C). In contrast, the taxonomic profile of the metagenome showed a larger contribution of bacterial reads and, in particular, of reads assigned to *Cyanobacteria* and *Bacteroidetes* (Fig. 1A). Because of the use of strand-specific RNA-seq libraries, we confidently identified both intergenic (it)sRNA, located between coding regions, and antisense (a)sRNA, overlapping with their putative target (Table 1). We found 3 times more itsRNAs in the *Archaea* than in the *Bacteria*, whereas asRNAs were more abundant in the *Bacteria* and more often associated with members of the *Cyanobacteria* (38%) and *Bacteriodetes* (15%) (Table 1; Fig. 1D and E). We also found 79 ncRNAs that belong to 6 known families of RNAs present in the Rfam database (Fig. S2D; Data Set S1) (23), validating our experimental and computational approaches. This database is a collection of RNA families, each represented by multiple sequence alignments, consensus secondary structures, and covariance models. Of the Rfam-conserved ncRNAs, 70% were assigned to archaea and included RNaseP RNAs, signal recognition particle RNAs (SRP RNAs), and tRNAs. Of the Rfam-conserved bacterial ncRNAs, most were from SRP RNAs and tRNA conserved families. In addition, a cobalamin riboswitch and the regulatory sRNA, CyVA-1, were detected in low abundance in the halite *Cyanobacteria*. We also found 3 ncRNAs (4%) from eukarya, a tRNA, a U4 spliceosomal RNA, and a RNase for mitochondrial RNA processing (MRP). Using blastn analysis (maximum E value of 1E−3, sequence similarity of 70% or more, coverage of 50% or more), we discovered another 155 ncRNAs that were conserved in the NCBI nt database, with 60% from archaea and 40% from bacteria (Table 1). The majority were asRNAs (109), with only 44 itsRNAs. The conserved asRNAs most highly expressed (standardized transcripts per million [TPM] > 100) were all SPR RNAs in haloarchaea that were not found in the Rfam database. Of the conserved itsRNAs, we identified 3 tRNAs, 13 SRP RNAs, and 22 ncRNAs that were found in the genome of multiple species, all *Halobacteria*, but with no function assigned. The most highly expressed and conserved itsRNAs (standardized TPM > 100; 13 ncRNAs) were SRP RNAs not included in the Rfam database.

Another validation of our findings was the presence of canonical promoter elements upstream of archaeal itsRNAs, suggesting that they were indeed bona fide transcripts that could recruit basal transcription factors (see Fig. S3A). We did not find significant promoter elements upstream of the bacterial itsRNAs, which might reflect the diversity of promoter elements across the various bacterial taxa we identified in the halite community. In contrast, no promoter elements were identified in the upstream regions of asRNAs from both domains of life.

10.1128/mSystems.00584-19.3 FIG S3 (A) Regulatory regions for asRNAs and itsRNAs and for archaea and bacteria. (B) Expression level of itsRNAs, asRNAs, and protein-encoding genes normalized by contig abundance. Download FIG S3, TIF file, 1.5 MB. Copyright © 2020 Gelsinger et al. 2020 Gelsinger et al. This content is distributed under the terms of the Creative Commons Attribution 4.0 International license.

When looking at the expression levels of all itsRNAs normalized to contig abundances, we found that they were similar for both the 2016 and 2017 samples and slightly higher than that of the asRNAs, whereas the expression profile of the asRNAs was more variable across samples for both years (Fig. S3B). Remarkably, the expression levels of itsRNAs and asRNAs for both years were 2-fold higher than that of protein-encoding genes. Whereas there is an inherent bias in our approach to identifying sRNAs at the community level (coverage threshold in SnapT) compared to that for protein-encoding genes, this finding strongly indicates potential functional relevance for a number of these sRNAs.

We experimentally validated several sRNAs using reverse transcription-PCR (RT-PCR) with environmental and enrichment cultures (see Table S2). Enrichments were performed with several media containing high (25%) and relatively low (18%) salt and various combinations of carbon sources. Amplicon sequencing of the enrichments revealed that high salt and diverse carbon sources resulted in a higher diversity of taxa, although haloarchaea dominated in all enrichments (see Fig. S4). All validated sRNAs belong to haloarchaea except for one from *Cyanobacteria*. Sequences of the PCR products confirmed that they were sRNAs and validated our computational approach.

10.1128/mSystems.00584-19.4 FIG S4 Taxonomy distributions of halite enrichment cultures showing the relative abundance (%) of bacterial and archaeal taxa as a function of the culture medium. Download FIG S4, TIF file, 2.2 MB. Copyright © 2020 Gelsinger et al. 2020 Gelsinger et al. This content is distributed under the terms of the Creative Commons Attribution 4.0 International license.

10.1128/mSystems.00584-19.10 TABLE S2 Experimentally validated itsRNAs. Download Table S2, PDF file, 0.1 MB. Copyright © 2020 Gelsinger et al. 2020 Gelsinger et al. This content is distributed under the terms of the Creative Commons Attribution 4.0 International license.

### Relationship with target genes and putative function of community asRNAs.

Using our strand-specific RNA-seq data, we were able to identify the overlap positions of asRNAs to their antisense transcripts. We found that, in both *Archaea* and *Bacteria*, the majority of asRNAs start within the span of their cognate gene and end near the 5′ end of its mRNA. In both domains, there is also an enrichment for asRNA-mRNA overlaps near the 5′ end of the mRNA (Fig. S2E). A similar trend was previously reported in two species of archaea (21, 24).

**FIG 2:**
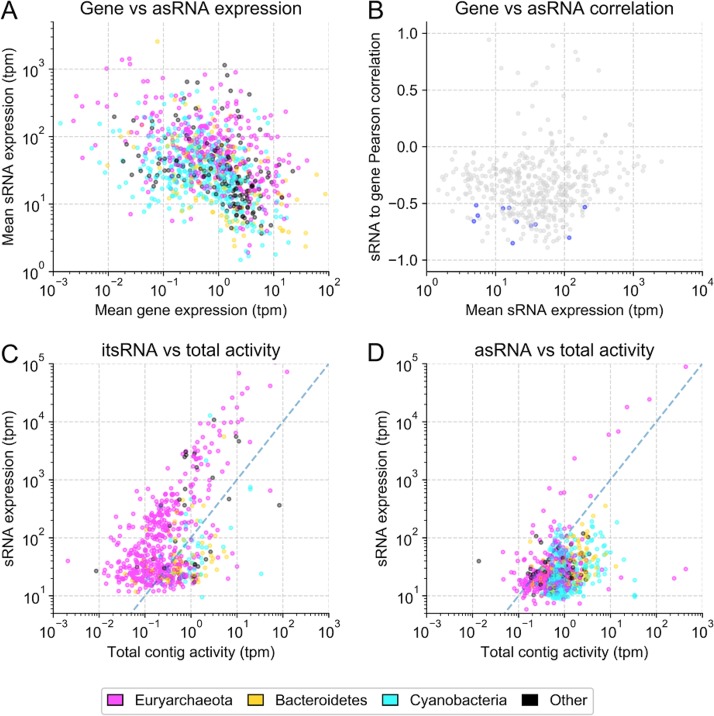
sRNA expression levels. (A) asRNAs and their putative targets (mean expression levels of all replicates) (TPM). (B) Pearson correlations for expression levels of asRNAs and their putative mRNA targets across all the replicates, with significant correlations (*P* < 0.01) highlighted in blue. Average expression of itsRNAs (C) and average expression of asRNAs (D) over the average expression of the contigs on which they are found. Dashed lines are added for simpler visual interpretation and represent a 1:1 ratio of contig activity to sRNA expression.

We compared the expression levels of asRNAs with those of their putative target genes and found that highly expressed asRNAs were associated with lowly expressed genes (Fig. 2A). Of gene pairs with asRNA expression >100 TPM and gene expression <0.1 TPM, most where from haloarchaea (77%), with 12% of *Cyanobacteria*, and 11% of other bacteria (*Bacteriodetes* and Acinetobacter) (Data Set S1). Gene functions were enriched for transport (16%) and cell membrane/wall metabolism (5%), while most were hypothetical proteins (44%). Of the genes potentially negatively regulated by their cognate asRNAs, we found an archaeal regulator of the IclR family and potassium uptake protein TrkA. Only 2 asRNAs with high expression levels (>100 standardized TPM) were associated with genes with relatively high expression levels (>1 standardized TPM), while still being negatively correlated (Fig. 2A). The corresponding genes encoded an iron complex outer membrane receptor protein from *Salinibacter* and an ABC-type sodium efflux pump permease subunit from a *Halobacteria*. When applying a stringent cutoff, we found 9 statistically significant and negatively correlated asRNA-gene pairs (Fig. 2B and S5A). Four were from *Bacteroidetes*, 4 from *Halobacteria*, and 1 from an unidentified bacterium. At the functional level, transport systems, and in particular, iron transport systems, were particularly enriched (Data Set S1). In contrast, we did not find any significant positive regulation between asRNAs and their cognate genes. When adjusted for the carrying organism’s abundance, expressed as the average RNA read coverage of the contigs, we found that, overall, itsRNAs were more highly expressed than asRNAs (Fig. 2C and D). Highly expressed sRNAs, for both types, were mostly carried by haloarchaea.

10.1128/mSystems.00584-19.5 FIG S5 PCA plots of Pearson correlations in expression levels between asRNAs and their putative mRNA targets across all the replicates, color coded by taxa (A), and expression levels of annotated genes from the metatranscriptome for all samples (B). Download FIG S5, TIF file, 0.5 MB. Copyright © 2020 Gelsinger et al. 2020 Gelsinger et al. This content is distributed under the terms of the Creative Commons Attribution 4.0 International license.

### Differential expression of itsRNAs at the community level and target prediction.

**FIG 3:**
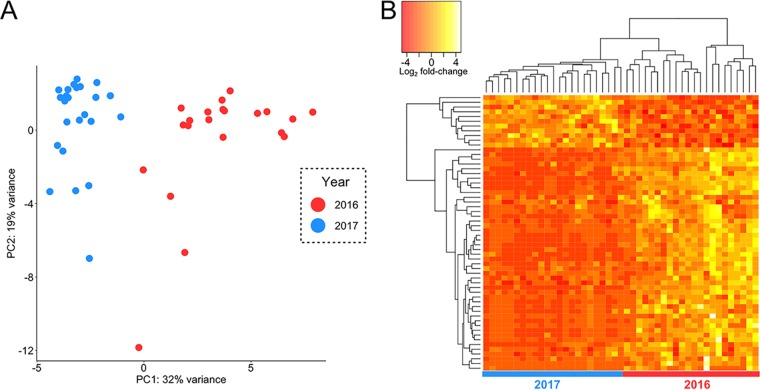
itsRNA differential expression. (A) Principal-component analysis (PCA) plot showing itsRNA expression levels clustered by year. (B) Heat map of log_2_-transformed fold changes for the top 50 significantly differentially expressed itsRNAs; each row is an itsRNA and each column a sample collected in 2016 or 2017.

**FIG 4:**
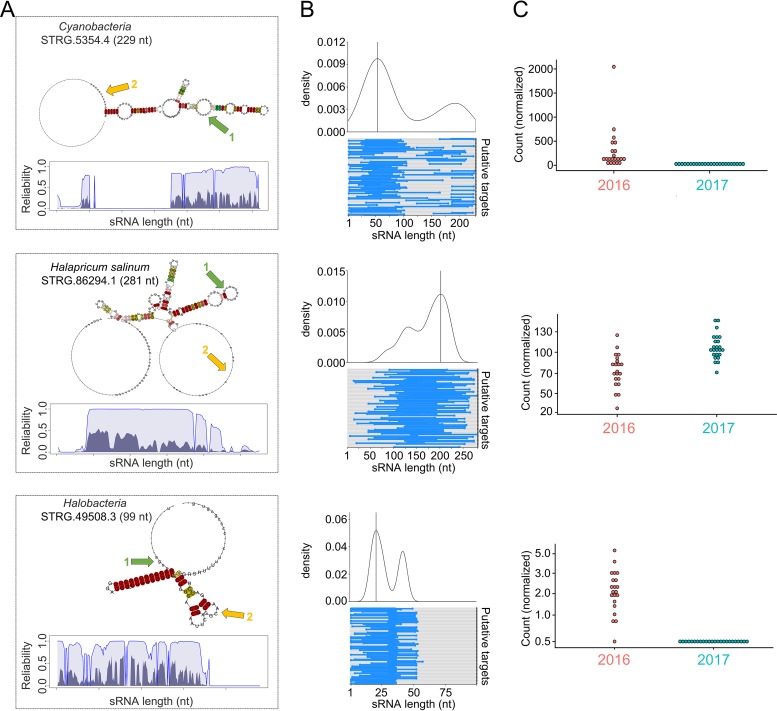
Predicted structure, target identification, and expression levels for selected differentially expressed itsRNAs. (A) Two-dimensional (2D) layout of consensus structures with base-pair coloring showing sequence and structure conservation and interactions peaks (green and yellow arrows); STAR profile plots with dark regions indicating structure reliability, light regions representing sequence reliability, and thin lines showing the combined column reliability as computed by LocARNA-P. (B) Interaction plots of itsRNAs and their predicted targets. The top graphs are density plots calculated from the top 100 putative targets, and on the bottom are dumbbell plots of interactions (blue dumbbells) along the length of the itsRNA for the top 100 predicted mRNA targets; interaction peaks are shown in green and yellow in the predicted structures; (C) Expression levels represented as normalized counts for each itsRNA in 2016 and in 2017 across all samples.

Analysis of itsRNAs expression levels showed a clear separation between the 2016 and 2017 samples (Fig. 3A), which was confirmed by the analysis of metatranscriptomic expression levels of annotated genes from the metagenome (Fig. S5B). We carried out a differential expression analysis and found that 109 (18%) of the regulatory itsRNAs were significantly differentially expressed (false-discovery rate [FDR] < 5%) between samples collected in 2016 and 2017 (Fig. 3 and Data Set S1), 3 and 15 months after a major rain event in the desert, respectively (14). Of these, 72% were annotated as archaea and 28% as bacteria, and 16 were conserved in multiple genomes (14 from *Halobacteria* and 2 from *Cyanobacteria*). Conservation of differentially expressed itsRNAs allowed for structure modeling and, when high-quality metagenome-assembled genomes (MAGs; >70% completion and <5% contamination) were available from the metagenome, target prediction (Fig. 4 and S6). Several nondifferentially expressed itsRNAs were also conserved, providing additional opportunity for structure prediction; these included itsRNAs from *Halococcus* (STRG.48671.1; 69 nucleotides [nt]), Halobellus limi (STRG.136887.1; 209 nt), and a member of the *Nanohaloarchaea* (STRG.4577.1; 266 nt) (Fig. S6A).

10.1128/mSystems.00584-19.6 FIG S6 (A) Additional structure prediction for highly expressed and differentially expressed itsRNAs. (B) Predicted structure, target identification, and expression levels for differentially expressed itsRNA STRG.5356.1 from a cyanobacterium. Download FIG S6, TIF file, 2.5 MB. Copyright © 2020 Gelsinger et al. 2020 Gelsinger et al. This content is distributed under the terms of the Creative Commons Attribution 4.0 International license.

All predicted structures displayed stem-loop regions that had high sequence conservation (light purple regions on sequence-structure-based alignment reliability [STAR] profile plots) and high structure conservation (dark purple), with line plots representing the reliability of the predictions as calculated by LocaRNA (Fig. 4 and S6B). Density plots combined with dumbbell plots were used for visualizing predicted interactions between itsRNAs and their putative targets, using IntaRNA data from the top 100 most reliable interaction predictions with the lowest free energy of hybridization (25) (Fig. 4). High confidence assignments were obtained for 4 differentially expressed itsRNAs from *Cyanobacteria*, Halapricum salinum, and a member of the *Halobacteria* (Data Set S1) More than one interaction peak was derived from density plots; peak 1 (green) corresponded to the highest interaction density, which mapped to loop regions in the itsRNA secondary structure with high sequence and structure conservation and was thus a confident assignment as an interaction region, whereas peak 2 (yellow) was a less confident assignment structurally despite high interaction density (Fig. 4 and S6B).

Using this information, we identified the most probable targets for *Cyanobacteria* STRG.5354.4 candidate itsRNA (229 nt). This itsRNA was conserved as a 6S regulatory RNA in the Rfam database, which, in *Bacteria*, is found to inhibit transcription by binding directly to the housekeeping holoenzyme form of RNA polymerase (26). Of the top 50 most probable targets for STRG.5354.4, which were those with the lowest free energy of hybridization between itsRNA and targets, were cation/H^+^ antiporters (shown to be involved in osmoregulation [27]), a PleD family two-component response regulator, the photosystem I PsaB protein, chemotaxis transducers, and proteins involved in energy metabolism. Most probable targets for differentially expressed itsRNA, STRG.86294.1 (281 nt) from Halapricum salinum included various transporters and putative membrane and cell wall-associated proteins; notably, an ammonium transporter (Amt family), an alkanesulfonate monooxygenase SsuD from a gene cluster expressed under sulfate or cysteine starvation (28), and several proteins involved with cofactors and vitamin metabolism. Predicted targets with the lowest free energy of hybridization for STRG.49508.3 candidate itsRNA (99 nt) from *Halobacteria* were elongation factor 1-alpha, which promotes the GTP-dependent binding of aminoacyl-tRNA to the A-site of ribosomes during protein biosynthesis, several ribosomal proteins, and hypothetical proteins. Target prediction for *Cyanobacteria* STRG.5356.1 candidate itsRNA (242 nt) included molecular chaperones (DnaK and DnaJ classes), a cell division protease FtsH, and several uncharacterized proteins.

## DISCUSSION

The roles of regulatory sRNAs have been extensively studied in *Bacteria* and, to a lesser extent, in archaeal model systems (1, 5), but to date, only four studies have reported the discovery of sRNAs in microbial communities. In one study, Shi et al. (6) used metatranscriptomic data to identify unique microbial intergenic sRNAs in the ocean’s water column. In a second study, Bao et al. (7) revealed extensive antisense transcription in the human gut microbiota, also using metatranscriptomic data sets. In the last two studies, Hou et al. (9) conducted a survey of transcription start sites (TSS) and identified a small number sRNA TSS, while Duran-Pinedo et al. (8) carried out an extensive study of intergenic sRNAs 45 m deep in the northern Red Sea and focused on those that were conserved in the Rfam database. Efforts have also been made to mine publicly available databases for sRNA discovery (29), but this was still addressing the role of sRNAs in single microorganisms. Each of these studies was limited to one type of sRNA (intergenic or antisense) usually due to technical limitations (i.e., sequencing technology, library preparations, etc.). Through the combination of strand-specific RNA sequencing and the development of the first microbial sRNA identification pipeline, SnapT, we were able to comprehensively identify all sRNAs in an extremophilic microbial community. This combination of technologies allowed for a highly resolved view of sRNA-mediated regulation from multiple trophic levels in the community, from primary producing cyanobacteria to the dominant heterotrophic haloarchaea.

One major difficulty in obtaining metatranscriptomic data from natural microbial communities, in particular, from extreme environments, is the small amount of biomass that can be collected, resulting in low RNA yields (14). This, in turn, prevents attempts at ribodepletion, resulting in a decreased number of non-rRNA reads available for analysis. Nevertheless, using SnapT, a flexible pipeline to process metagenomics and metatranscriptomic data, we report the discovery of hundreds of diverse sRNAs from an extremophilic community inhabiting halite nodules in the Atacama Desert. In the process, we applied extensive quality control with coverage thresholding, correction for contig edge misannotation, and the removal of potential protein-coding RNAs through sequence and homology searches. While this approach might potentially result in false negatives and may bias our findings toward the most highly expressed sRNAs in the community, it also ensured the robustness of our sRNA predictions by minimizing the number of false positives. The identification of ncRNAs in the halite community that belongs to the Rfam database (23), together with experimental validation of several sRNAs with environmental and enrichment cultures, substantiated our analytical approach. Additionally, expression levels of sRNAs 2-fold higher than that of protein-encoding genes strongly indicate potential functional relevance for a number of these sRNAs.

The taxonomic composition of the halite sRNAs matched that of the community’s metatranscriptomic profile, reflecting the contribution of the most active members, including *Cyanobacteria*, *Bacteriodetes*, and several *Halobacteria*. We found significantly more itsRNAs in the *Archaea* than in the *Bacteria*, and the trend was reversed for the asRNAs. This novel finding is representative of published work in model organisms, where a wide range of sRNAs has been found so far in prokaryotes, from less than a dozen to more than a thousand per genome (see Fig. S7 in the supplemental material) (1, 5).

10.1128/mSystems.00584-19.7 FIG S7 sRNA distribution per genome of model organisms from previous studies from the literature. Download FIG S7, TIF file, 0.2 MB. Copyright © 2020 Gelsinger et al. 2020 Gelsinger et al. This content is distributed under the terms of the Creative Commons Attribution 4.0 International license.

Antisense sRNAs overlap their putative targets, providing insights into their functional role (4). In the halite community, we found that asRNA expression levels were negatively correlated with those of their putative targets, with highly expressed asRNAs overlapping lowly expressed protein-encoding genes. A similar trend was reported in the haloarchaeon H. volcanii when investigating oxidative stress-responsive sRNAs, and most of the putative targets were transposase genes (21). Putative target gene functions in our study were mostly from haloarchaea and enriched for transport systems, cell membrane, and cell wall metabolism, with a large number of hypotheticals. Of particular interest was an archaeal IcIR transcription regulator; these regulators are known to be involved in diverse physiological functions, including multidrug resistance, degradation of aromatics, and secondary metabolite production (30), and are distributed in a wide range of prokaryotes, including archaea (31). Also of interest was a Trk potassium uptake system, also found in both bacteria and archaea and essential for the maintenance of high intracellular potassium in salt-in strategists (32). Salt-in strategists accumulate KCl to balance the high osmotic pressure of their environment, hence the need to actively pump potassium into the cell. In contrast, we did not find any significant positive regulation between asRNAs and their cognate genes (upregulation of both), which might be due to the inherent quality of our data set, i.e., no ribodepletion and heterogeneity across replicates (14). Alternatively, it might also reflect promiscuous transcription processes as argued when considering the functionality of asRNAs (33). Other arguments in favor of spurious transcription were the size distribution for asRNAs found in the halite community, which was significantly larger than that of itsRNAs, low expression level when adjusted for organism abundance compared to that of itsRNAs, and the absence of canonical regulatory elements in the upstream regions of asRNAs. However, we also found putative target functions that reflected the environmental challenges faced by members of this extremophile community, such as osmoregulation and nutrient uptake, indicating that these asRNAs might indeed regulate fundamental biological functions at the community level.

We previously showed that the halite community dramatically shifted its taxonomic and functional composition after a major rain event in 2015, and while it recovered at the functional level in 2017, 15 months after the rain, members of the communities were permanently replaced (14). Here, we found that 18% of the halite community itsRNAs were significantly differentially expressed (FDR < 5%) between samples collected in 2016 and 2017 (3 and 15 months after the rain, respectively), potentially indicating a transcriptional response to changes in environmental conditions. Intergenic sRNAs are of particular interest because they can target multiple genes, including key transcription factors and regulators (3). As a consequence, a single sRNA can modulate the expression of large regulons and thus have a significant effect on metabolic processes (5). However, they do not overlap their target genes or bind their target mRNAs with perfect complementary, which makes finding targets for these sRNAs very challenging without genetic tools (1).

To solve this problem at the community level, we focused on itsRNAs that were conserved and for which we could perform structural prediction. The intersection of this small subset of sRNAs with high-quality MAGs that could be used as reference genomes yielded confident target predictions for 4 differentially expressed itsRNAs, giving insights into metabolic functions potentially regulated by sRNAs at the community level. These included transporters, particularly, those related to osmotic stress, nutrient uptake, and starvation, and pathways for chemotaxis and energy production and conversion. These pathways reflect the environmental challenges members of the halite communities are subjected to, including osmotic adjustments to climate perturbation (14) and competition for nutrients in a near-closed system with primary production as the major source of organic carbon (16). Using the genomic context of sRNAs from the ocean’s water column microbial communities, Shi et al. (6) reported similar metabolic functions, underlying the specific regulatory needs for natural communities. In contrast, genes with antisense transcription to asRNAs identified in the human gut microbiome were mostly transposase genes, with a small component of bacterial housekeeping genes (7). It important to note that no computational target prediction, using sRNA conserved predicted structure, was reported in either study. Our ability to predict *de novo* targets for sRNAs drastically increases the scale of regulatory potential we can map to a microbial community. Target prediction is entirely reliant on high-quality MAGs and gene annotation, which we have successfully performed through method development (14). Taking this together, we suggest that extremophilic communities, including the halite communities, can be used as model systems to study sRNA dynamics in a natural environment.

Regulation of transcription by 6S sRNA has been shown to increase competitiveness and long-term survival in bacteria (26), suggesting an important role for *Cyanobacteria* candidate sRNA STRG.5354.4, identified as a 6S sRNA. Because of high RNA-seq coverage of the *Cyanobacteria* MAGs, we were able to show that 40% of the top 50 targets for sRNA STRG.5354.4 were differentially regulated and more highly expressed in 2016, suggesting positive regulation by this sRNAs of its putative targets. Transcriptional factors and regulators were also found as putative targets of differentially regulated itsRNAs in the halite community, underlying the capacity of microbial sRNAs to modulate the expression of large regulons (1, 3, 34). Finally, a candidate itsRNA from the *Halobacteria* had several predicted targets associated with ribosomal proteins and proteins involved in translation processes. This finding, together with those from a recent study in H. volcanii (35), supports the idea of sRNA modulation of protein biosynthesis in the *Archaea*. A potential framework for mechanisms for sRNA regulation of translation might be provided by a report on the modular translation subsystems in the haloarchaeon Halobacterium salinarum that might selectively translate a subset of the transcriptome under specific growth conditions (36).

In this study, we characterized the taxonomic and functional landscape of sRNAs across two domains of life in an extremophilic microbial community, demonstrating that asRNAs and itsRNAs can be reliably identified from natural environmental communities. This is essential because sRNAs play essential roles in gene regulation across the 3 domains of life, but most sRNA studies have only been conducted with single organisms. Microorganisms do not live by themselves in the natural environment: they are found in communities, and if we want to understand the molecular mechanisms underlying community stress responses, it is essential to address the role of sRNAs in those regulatory processes. To facilitate this work, we built a flexible pipeline, SnapT (https://github.com/ursky/SnapT), leveraged by our expertise of sRNA biology in a model halophilic archaeon and which is available to use with metatranscriptomic data from any community. We demonstrated that we could perform target prediction and correlate expression levels between itsRNAs and predicted target mRNAs, paving the way for novel discoveries at the community level. While additional work with enrichment cultures remains to be conducted to fully characterize the functional roles of sRNAs from the halite community and their mechanism of action, these differentially expressed sRNAs for which we found putative targets show the power of community-level culture-independent approach analysis for gene regulation processes.

## MATERIALS AND METHODS

### Sample and weather data collection and nucleic acid extraction.

Halite nodules were harvested in Salar Grande, an ancient evaporated lake in the Northern part of the Atacama Desert (37) in February 2016 and 2017, 3 and 15 months after a major rain event, respectively (14). All nodules were harvested within a 50-m^2^ area as previously described (37). The colonization zone of each nodule was grounded into a powder, pooling 1 to 3 nodules until sufficient material was collected, and stored in the dark under dry conditions until DNA extraction in the lab. Samples used for RNA were stored in RNAlater at 4°C until RNA extraction in the lab within 14 days of collection. Genomic DNA was extracted with the DNeasy PowerSoil DNA extraction kit (Qiagen) as previously described (16, 37) (Qiagen). Total RNA was extracted from the fixed samples by first isolating the cells, gradual dissolving the salt particles as previously described (16, 37), and lysing them by mechanical bead beating with the RNAeasy PowerSoil RNA extraction kit (Qiagen). Total RNA was then extracted from the lysate with a Quick-RNA miniprep kit (RNA > 17 nt) (Zymo Research). We obtained 10 to 100 ng of RNA/g of grounded halite. RT-PCR was used to validate the absence of contaminating DNA in the total RNA used for RNA-seq libraries with 16S rRNA primers 515F/926R (18).

### Library preparation.

Whole-genome sequencing libraries were prepared using the KAPA HyperPlus kit (Roche) as previously described (14) and sequenced with paired 150-bp reads on the HiSeq 2000 platform at the Johns Hopkins Genetic Resources Core Facility (GRCF). Total RNA-seq libraries were prepared with the SMARTer Stranded RNA-seq kit (38), using 25 ng of RNA input and 12 cycles for library amplification, as previously described (18). We sequenced 21 libraries from replicate samples from 2016 and 24 libraries from replicate samples from 2017 (see Table S1 in the supplemental material).

### Metagenomic sequence processing and MAG recovery.

The demultiplexed shotgun metagenomic sequencing reads were processed with the complete metaWRAP v0.8.2 pipeline (39) with recommended databases on a UNIX cluster with 48 cores and 1024 GB of RAM available. This study used the publicly available metagenomic assembly, annotation, and metagenome-assembled genomes (MAGs) from previous work (14). MAGs with minimum completion of 70% and maximum contamination of 5%, as determined with CheckM (40), were used in this study. Detailed scripts for the entire analysis pipeline can be found at https://github.com/ursky/timeline_paper.

### SnapT for sRNA community identification.

An analytic pipeline, SnapT for Small ncRNA Annotation Pipeline for (meta)Transcriptomic data, was adapted from our previous work (21) to find, annotate, and quantify intergenic and antisense sRNA transcripts from transcriptomic or metatranscriptomic data. In brief, *de novo* transcripts were assembled from RNA reads mapped to the metagenomic assembly, and transcripts that could not be explained by any protein-coding region and did not encode peptides were extracted and further validated as sRNAs. Detailed scripts for the pipeline can be found at https://github.com/ursky/SnapT, and search criteria were as follows: intergenic transcripts were at least 30 nt away from any gene or open reading frame (ORF) on both strands; antisense transcripts were 30 nt away from any gene on their strand, but overlapped with a gene on the opposite strand by at least 10 nt; small peptides (<100 nt) were not counted as genes if they were encoded in a transcript that was more than 3 times their length; noncoding transcripts could not contain any reading frame greater than one-third of their lengths; predicted noncoding transcripts near contig edges were discarded, and the minimum distance to the edge of a contig was dynamically computed such that the tips of contigs were not statistically enriched in annotated ncRNAs; small ncRNAs were between 50 nt and 500 nt in length; sRNA transcripts could not have significant homology with any protein in the NCBI nr database (query cover > 30%, Bitscore > 50, E value < 0.0001, and identity > 30%) and with any tRNA, RNase P, or signal recognition particle (SRP) model in the Rfam noncoding RNA database.

### Taxonomic assignment and distribution of sRNAs.

The taxonomic origin of each annotated sRNA was taken to be that of the contig on which it lies. The taxonomy of each contig was estimated by taking the weighted average of the taxonomic assignment of the genes encoded on it, as determined through the JGI IMG functional and taxonomic annotation service (https://img.jgi.doe.gov/).

### Metatranscriptomic correlation and differential expression analysis.

We used a read count-based differential expression analysis to identify differentially expressed sRNA and mRNA transcripts. The program featureCounts (41) was used to rapidly count reads that map to the assembled RNA transcripts (described above) as previously described (21). To account for organism abundance changes (as opposed to true transcript changes), we normalized the transcript read counts to the total read counts from the contig on which the transcript lies. The read counts were then used in the R differential expression software package DESeq2 (42) to calculate differential expression by determining the difference in read counts between 2016 normalized read counts from 2017 normalized read counts. The differentially expressed RNAs were filtered based on the statistical parameter of false-discovery rate (FDR), and those that were equal to or les than an FDR of 5% were classified as true differentially expressed transcripts. We carried out differential expression analysis using a pairwise Wald test to find any possible differences between years (42). In parallel, normalized expression values were calculated using stringtie in transcripts per million (TPM). TPM of transcripts was normalized in the same way as read counts, except using contig TPM. TPM of transcripts was used for ranking of expression within samples as opposed to differential expression analysis.

### Regulatory element motif identification of sRNAs and structure and target prediction.

Fifty nucleotides upstream from the sRNA transcript start coordinates were searched for transcription motifs (BRE and TATA box for archaea and −35 and −10 consensus sequences for bacteria) using multiple sequence alignments, visualization with WebLogo, and motif searching with MEME (21). Conserved sRNAs were identified using blastn against the NCBI nt database. Secondary structures of conserved sRNAs were predicted using sRNAs that had an E value maximum of 1E−3, sequence similarity of 70% or more, and 50% or more coverage with a NCBI nt database blastn hit; a minimum of 14 alignments was used in the program LocARNA using global alignment settings (43). Lastly, putative targets were predicted for itsRNAs by searching for optimal sRNA-mRNA hybridization using the IntaRNA program with the “no seed” parameter (25) and the reference genes for each respective MAG. Targets were ranked by lowest *P* value. Expression levels for putative targets of antisense sRNAs were obtained from coexpression analysis of transcripts (21). The sRNA and putative target mRNA TPM expression values were tracked across the replicates, and the Pearson correlation was computed.

### Enrichment cultures.

Three types of culture medium were inoculated in triplicates with ∼2 g of grounded halite colonization zones and incubated at 42°C with shaking at 220 rpm (Amerex Gyromax 737) for 1 to 2 weeks. Cells were harvested by centrifugation, and nucleic acids were extracted as described above. The media were GN101 medium (44) containing 250 g of salt per liter and 10 g of peptone as carbon source, Hv-YPC medium (45) containing 250 g of salt per liter and 8.5 g of yeast extract, 1.7 g of peptone, and 1.7 of Casamino Acids as carbon sources, and IO medium containing 250 g of salt and the same carbon sources as the Hv-YPC medium. The taxonomic distributions of the cultures were obtained with 16S rRNA gene sequencing as previously described (14).

### sRNA validation.

Total RNA extracted from environmental samples and enrichment cultures was converted into cDNA using the SuperScript III first-strand synthesis system (Thermo Fisher) using 5 ng of input RNA. The cDNA was then amplified using 515F/926R 16S rRNA primers as previously described (18). Amplicons were sequenced using Sanger sequencing (GENEWIZ).

### Data availability.

Raw sequencing data are available from the National Center for Biotechnology Information under NCBI project identifier (ID) PRJNA484015. The metagenome coassembly and functional annotation are available from the JGI Genome Portal under IMG taxon OID 3300027982. Metatranscriptome data have been deposited in NCBI’s Gene Expression Omnibus and are accessible through GEO Series accession number GSE137164. Scripts for functional annotation, statistical analyses, differential expression, and figures are available at https://github.com/ursky/srna_metatranscriptome_paper.
