# Supplementary figures and images for: Regulatory Noncoding Small RNAs Are Diverse and Abundant in an Extremophilic Microbial Community
Source: mSystems. 2020 Feb 4;5(1):e00584-19. doi: 10.1128/mSystems.00584-19 (PMC7002113; doi:10.1128/mSystems.00584-19)

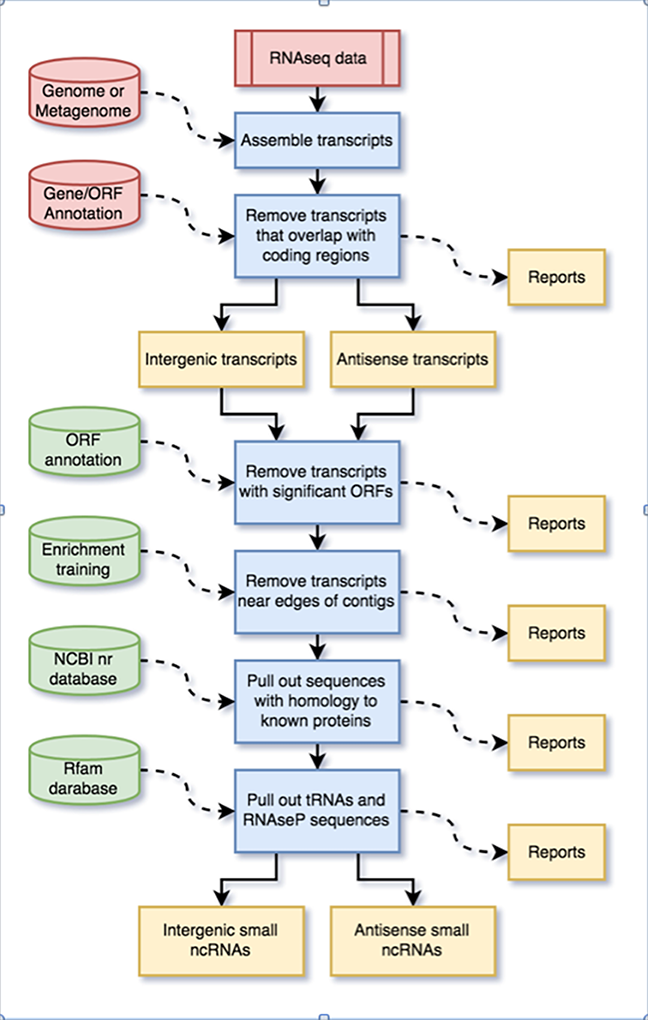

Supplement: FIG S1 [file mSystems.00584-19-sf001.tif]

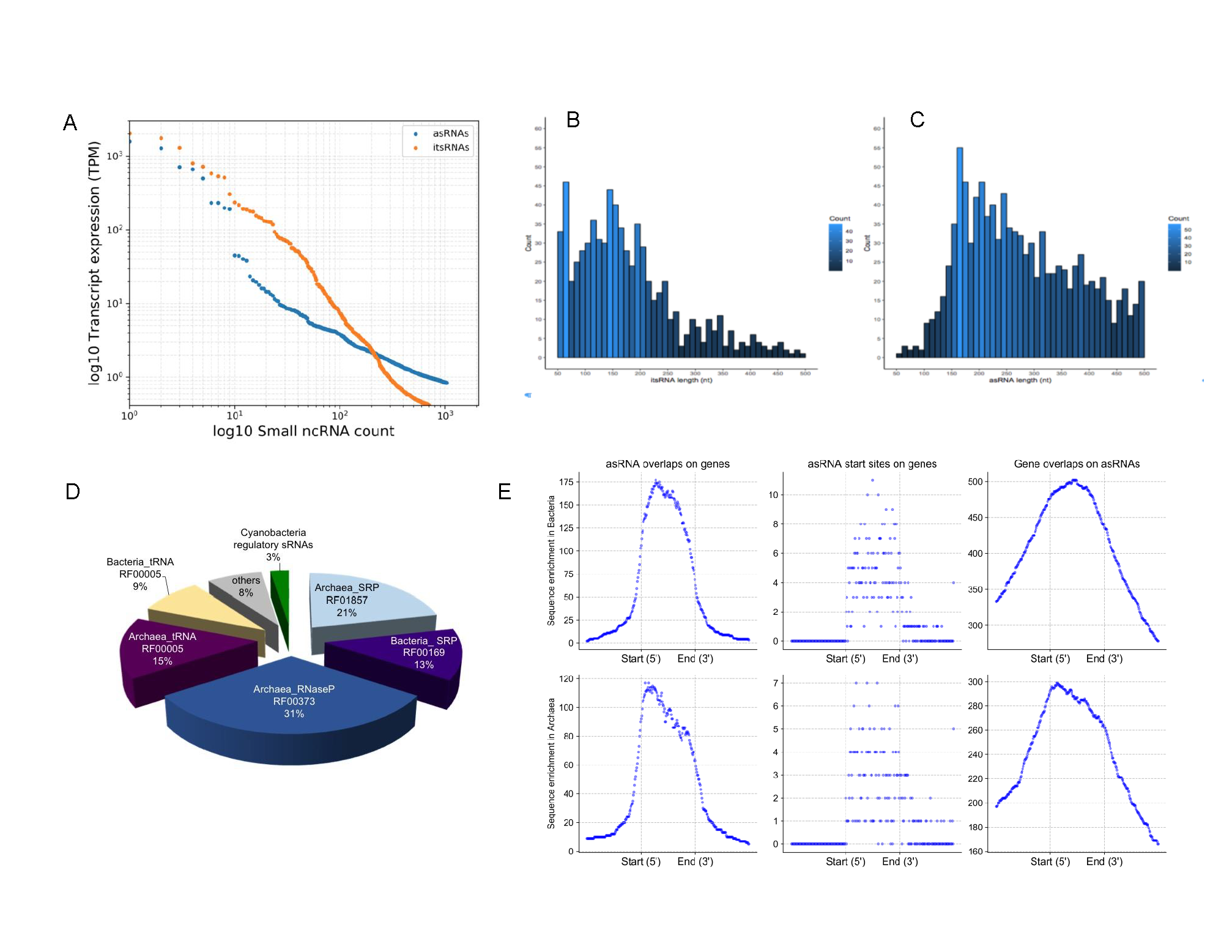

Supplement: FIG S2 [file mSystems.00584-19-sf002.tif]

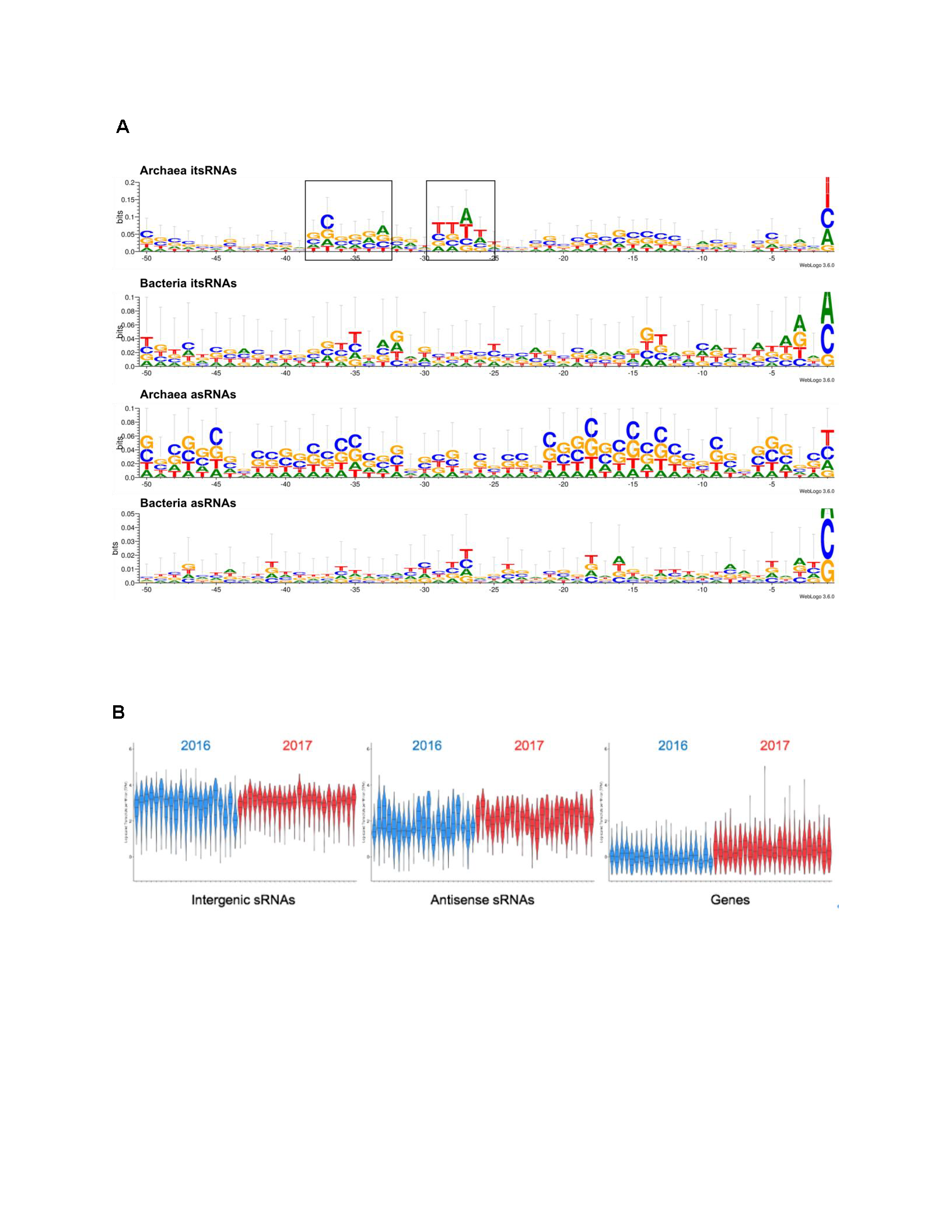

Supplement: FIG S3 [file mSystems.00584-19-sf003.tif]

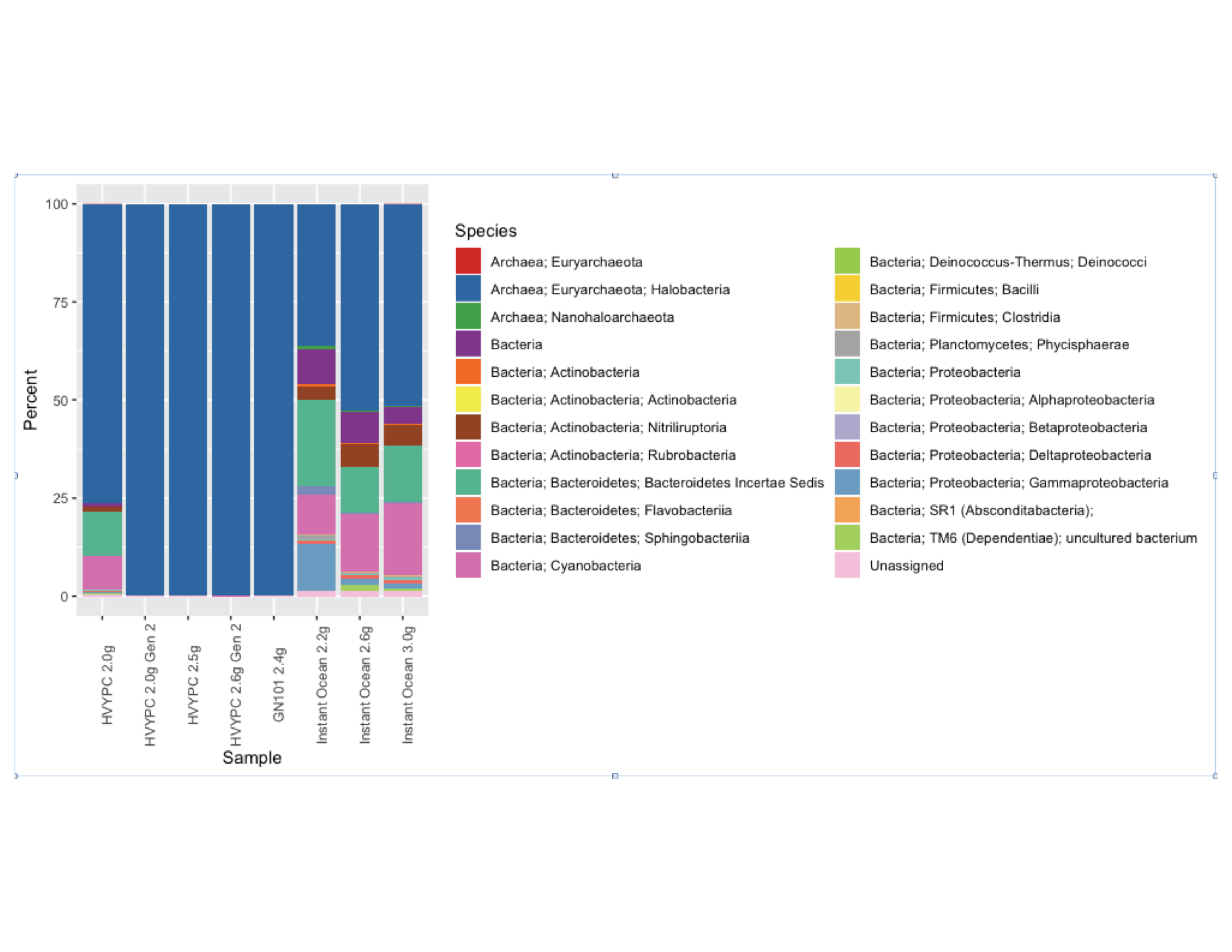

Supplement: FIG S4 [file mSystems.00584-19-sf004.tif]

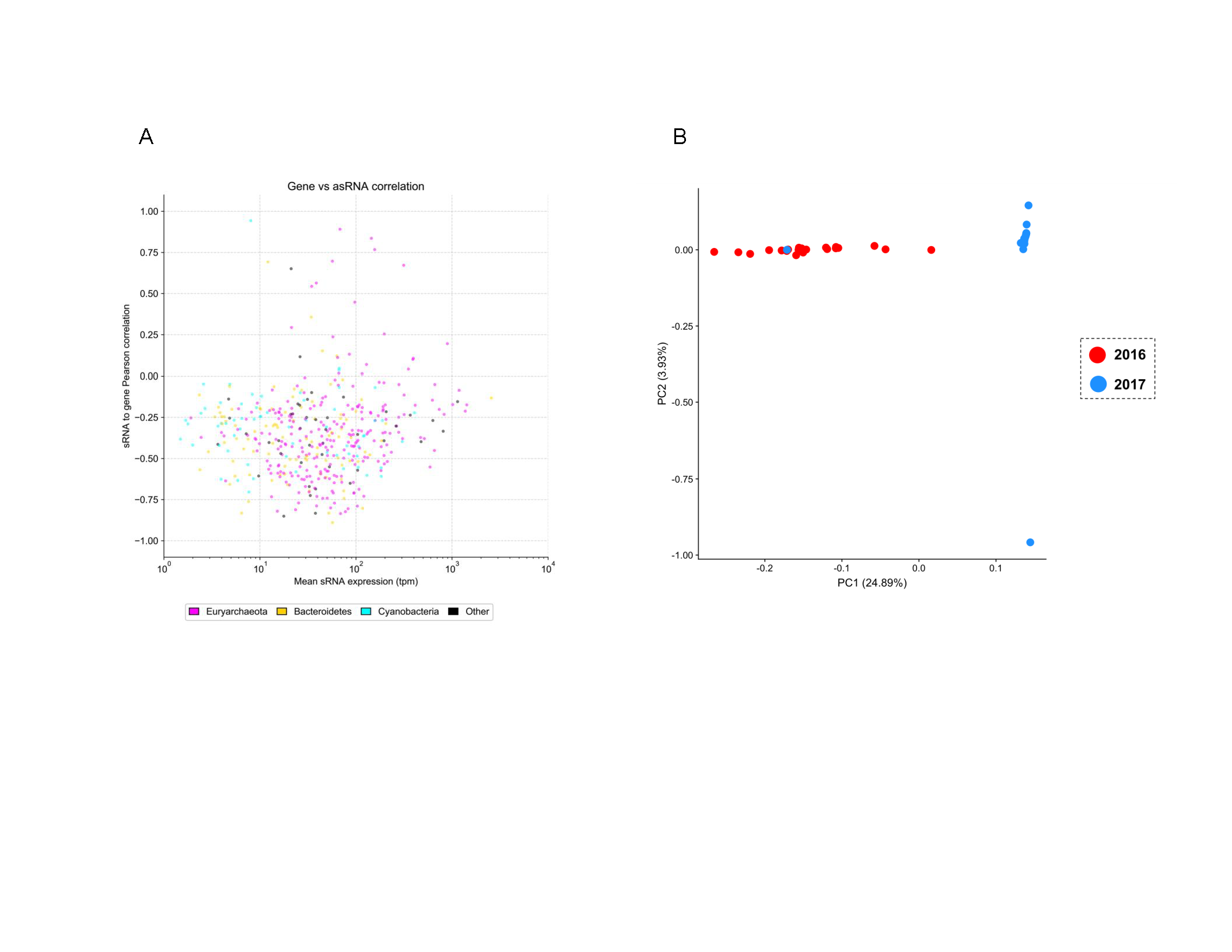

Supplement: FIG S5 [file mSystems.00584-19-sf005.tif]

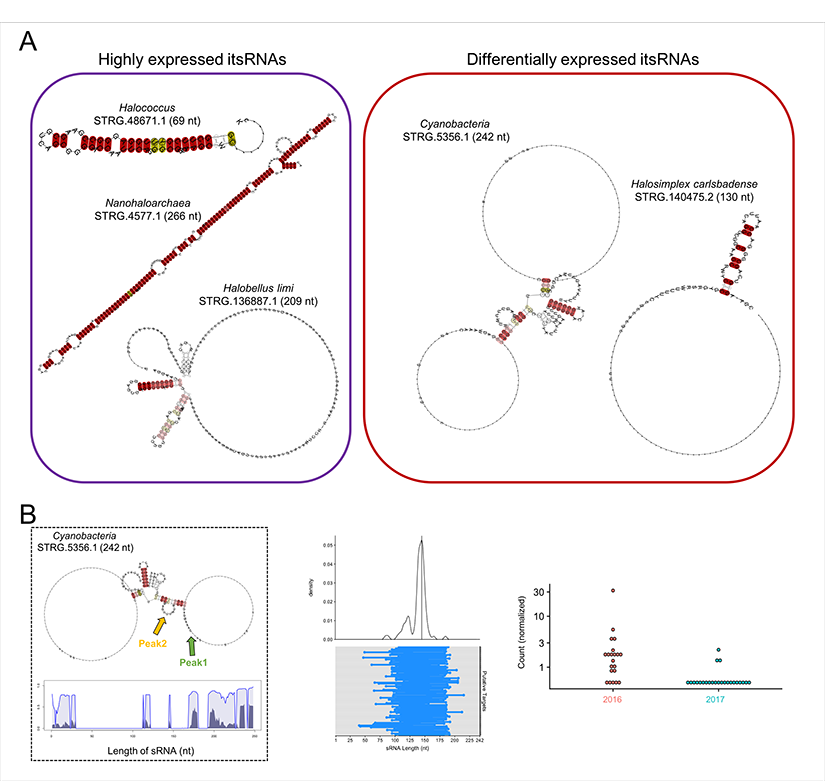

Supplement: FIG S6 [file mSystems.00584-19-sf006.tif]

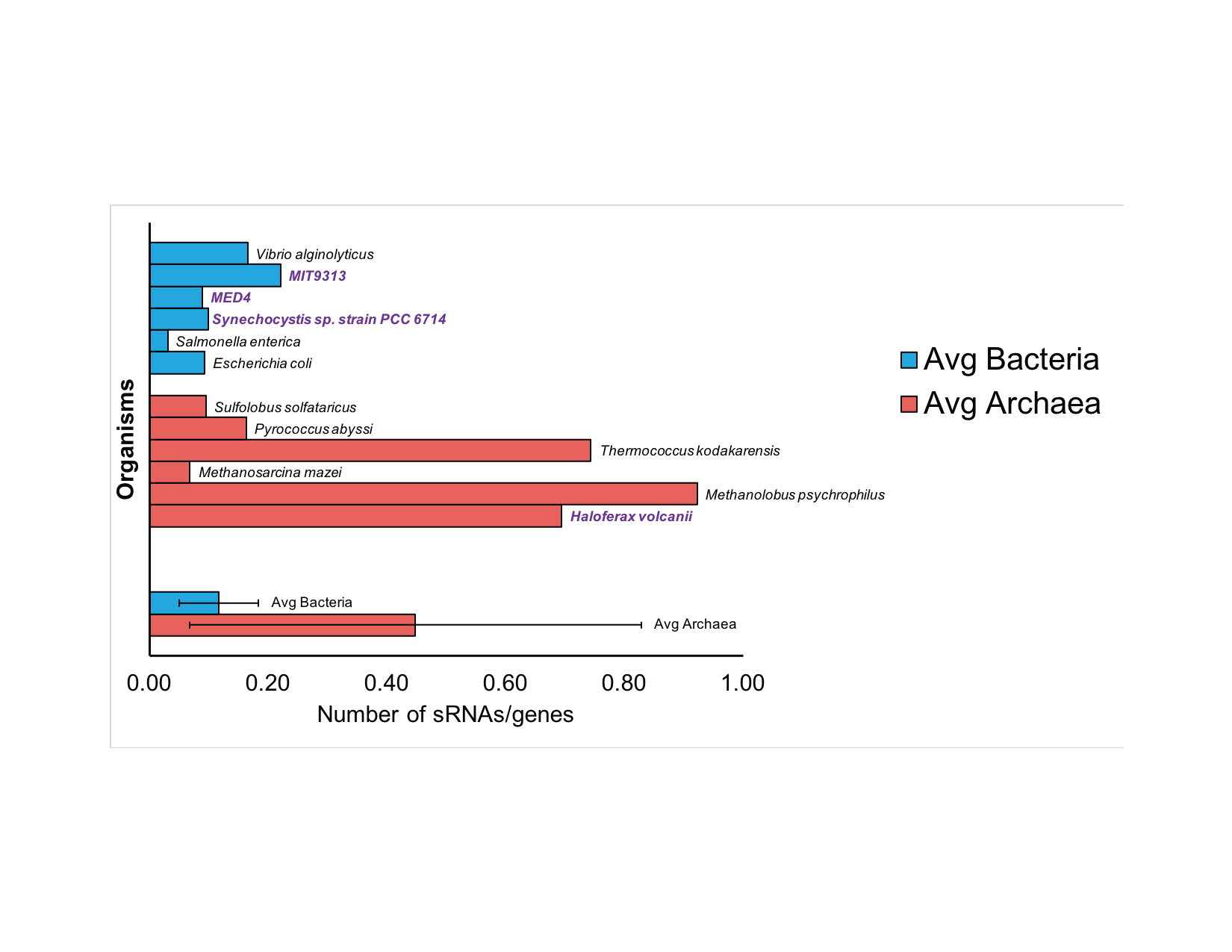

Supplement: FIG S7 [file mSystems.00584-19-sf007.tif]
