# Supplementary material for: Regulatory Noncoding Small RNAs Are Diverse and Abundant in an Extremophilic Microbial Community
Source: mSystems. 2020 Feb 4;5(1):e00584-19. doi: 10.1128/mSystems.00584-19 (PMC7002113; doi:10.1128/mSystems.00584-19)
Supplement: TABLE S1 [file mSystems.00584-19-st001.pdf]

Table S1: Samples collected and sequenced libraries

	Sequencing libraries						
**Sample**	**Forward read file**	**Reverse read file**	**Lattitude**	**Longitude**	**Elevation**	**Collection date**	**Collection time**
s1a1	s1a1_1.fastq	s1a1_2.fastq	20°57’12.006”S	70°1’10.5996”W	680m	8-Feb-16	1:00
s1a2	s1a2_1.fastq	s1a2_2.fastq	20°57’12.006”S	70°1’10.5996”W	680m	8-Feb-16	1:00
s1a3	s1a3_1.fastq	s1a3_2.fastq	20°57’12.006”S	70°1’10.5996”W	680m	8-Feb-16	1:00
s1b1	s1b1_1.fastq	s1b1_2.fastq	20°57’12.006”S	70°1’10.5996”W	680m	9-Feb-16	1:00
s1b2	s1b2_1.fastq	s1b2_2.fastq	20°57’12.006”S	70°1’10.5996”W	680m	9-Feb-16	1:00
s1b3	s1b3_1.fastq	s1b3_2.fastq	20°57’12.006”S	70°1’10.5996”W	680m	9-Feb-16	1:00
s9b1	s9b1_1.fastq	s9b1_2.fastq	20°57’12.006”S	70°1’10.5996”W	680m	9-Feb-16	9:00
s9b2	s9b2_1.fastq	s9b2_2.fastq	20°57’12.006”S	70°1’10.5996”W	680m	9-Feb-16	9:00
s9b3	s9b3_1.fastq	s9b3_2.fastq	20°57’12.006”S	70°1’10.5996”W	680m	9-Feb-16	9:00
s13a1	s13a1_1.fastq	s13a1_2.fastq	20°57’12.006”S	70°1’10.5996”W	680m	8-Feb-16	13:00
s13a2	s13a2_1.fastq	s13a2_2.fastq	20°57’12.006”S	70°1’10.5996”W	680m	8-Feb-16	13:00
s13a3	s13a3_1.fastq	s13a3_2.fastq	20°57’12.006”S	70°1’10.5996”W	680m	8-Feb-16	13:00
s13b1	s13b1_1.fastq	s13b1_2.fastq	20°57’12.006”S	70°1’10.5996”W	680m	9-Feb-16	13:00
s13b2	s13b2_1.fastq	s13b2_2.fastq	20°57’12.006”S	70°1’10.5996”W	680m	9-Feb-16	13:00
s13b3	s13b3_1.fastq	s13b3_2.fastq	20°57’12.006”S	70°1’10.5996”W	680m	9-Feb-16	13:00
s17a1	s17a1_1.fastq	s17a1_2.fastq	20°57’12.006”S	70°1’10.5996”W	680m	8-Feb-16	17:00
s17a2	s17a2_1.fastq	s17a2_2.fastq	20°57’12.006”S	70°1’10.5996”W	680m	8-Feb-16	17:00
s17a3	s17a3_1.fastq	s17a3_2.fastq	20°57’12.006”S	70°1’10.5996”W	680m	8-Feb-16	17:00
s17b1	s17b1_1.fastq	s17b1_2.fastq	20°57’12.006”S	70°1’10.5996”W	680m	9-Feb-16	17:00
s17b2	s17b2_1.fastq	s17b2_2.fastq	20°57’12.006”S	70°1’10.5996”W	680m	9-Feb-16	17:00
s17b3	s17b3_1.fastq	s17b3_2.fastq	20°57’12.006”S	70°1’10.5996”W	680m	9-Feb-16	17:00
s2017-9am1	s2017-9am1_1.fastq	s2017-9am1_2.fastq	20°57’12.006”S	70°1’10.5996”W	680m	20-Feb-17	9:00
s2017-9am2	s2017-9am2_1.fastq	s2017-9am2_2.fastq	20°57’12.006”S	70°1’10.5996”W	680m	20-Feb-17	9:00
s2017-9am3	s2017-9am3_1.fastq	s2017-9am3_2.fastq	20°57’12.006”S	70°1’10.5996”W	680m	20-Feb-17	9:00
s2017-9am4	s2017-9am4_1.fastq	s2017-9am4_2.fastq	20°57’12.006”S	70°1’10.5996”W	680m	20-Feb-17	9:00
s2017-9am5	s2017-9am5_1.fastq	s2017-9am5_2.fastq	20°57’12.006”S	70°1’10.5996”W	680m	20-Feb-17	9:00
s2017-9am6	s2017-9am6_1.fastq	s2017-9am6_2.fastq	20°57’12.006”S	70°1’10.5996”W	680m	20-Feb-17	9:00
s2017-9am7	s2017-9am7_1.fastq	s2017-9am7_2.fastq	20°57’12.006”S	70°1’10.5996”W	680m	21-Feb-17	9:00
s2017-9am8	s2017-9am8_1.fastq	s2017-9am8_2.fastq	20°57’12.006”S	70°1’10.5996”W	680m	21-Feb-17	9:00
s2017-9am9	s2017-9am9_1.fastq	s2017-9am9_2.fastq	20°57’12.006”S	70°1’10.5996”W	680m	21-Feb-17	9:00
s2017-9am10	s2017-9am10_1.fastq	s2017-9am10_2.fastq	20°57’12.006”S	70°1’10.5996”W	680m	21-Feb-17	9:00
s2017-9am11	s2017-9am11_1.fastq	s2017-9am11_2.fastq	20°57’12.006”S	70°1’10.5996”W	680m	21-Feb-17	9:00
s2017-9am12	s2017-9am12_1.fastq	s2017-9am12_2.fastq	20°57’12.006”S	70°1’10.5996”W	680m	21-Feb-17	9:00
s2017-9pm1	s2017-9pm1_1.fastq	s2017-9pm1_2.fastq	20°57’12.006”S	70°1’10.5996”W	680m	20-Feb-17	21:00
s2017-9pm2	s2017-9pm2_1.fastq	s2017-9pm2_2.fastq	20°57’12.006”S	70°1’10.5996”W	680m	20-Feb-17	21:00
s2017-9pm3	s2017-9pm3_1.fastq	s2017-9pm3_2.fastq	20°57’12.006”S	70°1’10.5996”W	680m	20-Feb-17	21:00
s2017-9pm4	s2017-9pm4_1.fastq	s2017-9pm4_2.fastq	20°57’12.006”S	70°1’10.5996”W	680m	20-Feb-17	21:00
s2017-9pm5	s2017-9pm5_1.fastq	s2017-9pm5_2.fastq	20°57’12.006”S	70°1’10.5996”W	680m	20-Feb-17	21:00
s2017-9pm6	s2017-9pm6_1.fastq	s2017-9pm6_2.fastq	20°57’12.006”S	70°1’10.5996”W	680m	20-Feb-17	21:00
s2017-9pm7	s2017-9pm7_1.fastq	s2017-9pm7_2.fastq	20°57’12.006”S	70°1’10.5996”W	680m	21-Feb-17	21:00
s2017-9pm8	s2017-9pm8_1.fastq	s2017-9pm8_2.fastq	20°57’12.006”S	70°1’10.5996”W	680m	21-Feb-17	21:00
s2017-9pm9	s2017-9pm9_1.fastq	s2017-9pm9_2.fastq	20°57’12.006”S	70°1’10.5996”W	680m	21-Feb-17	21:00
s2017-9pm10	s2017-9pm10_1.fastq	s2017-9pm10_2.fastq	20°57’12.006”S	70°1’10.5996”W	680m	21-Feb-17	21:00
s2017-9pm11	s2017-9pm11_1.fastq	s2017-9pm11_2.fastq	20°57’12.006”S	70°1’10.5996”W	680m	21-Feb-17	21:00
s2017-9pm12	s2017-9pm12_1.fastq	s2017-9pm12_2.fastq	20°57’12.006”S	70°1’10.5996”W	680m	21-Feb-17	21:00
