# Supplementary material for: Regulatory Noncoding Small RNAs Are Diverse and Abundant in an Extremophilic Microbial Community
Source: mSystems. 2020 Feb 4;5(1):e00584-19. doi: 10.1128/mSystems.00584-19 (PMC7002113; doi:10.1128/mSystems.00584-19)
Supplement: TABLE S2 [file mSystems.00584-19-st002.pdf]

Table S2: Experimentally validated itsRNAs

			RT-PCR amplicon with		
sRNA_ID	Taxonomy	Primer set	Environ. sample	Enrichment cultures	Sequence
STRG.134829.3	*Halothece*		17A2, 9B2	IO-YPC; GN101; Hv-YPC	> STRG.134829.3<br>AACCTTACGGTTTGTCCAAAT<br>TAACCTCTGTACCGGTAGTCC<br>ATCGGAAATTGACGAGCAAC<br>CCTGAATTGACAGGGTGTCC<br>AATTAAACTGGATGGAGATA<br>CGATGACTAGCCGTTAGGTG<br>GTCAGCCTGCTAACCCTCGAT<br>TTGAGTGGCGGAGAACCTGA<br>GTGATTAGGTTCTAGAATCTC<br>CCACCATAATCTTTGATTTGG<br>TGGTGAGAGT
STRG.66426.1	*Salinarchaeum*		17B2	IO-YPC; GN101; Hv-YPC	> STRG.66426.1<br>GGTCGGACTAGGCTGGGCGG<br>TTAGGCCCCGCTCCGACGCC<br>CGCAGTACGGTCTTCAGCGG<br>GGGCCGAACCCGGGGACGTC<br>CGGTCAGACCGGGACGGGCC<br>TCGGAAGCCAACGTCGAAGC<br>CTCGTCCCTCGGGGACGACG<br>GTCCACGGCGGTCGCCTGCA<br>GGGGCGCGTTGTCGTGGTTC<br>GTCGGCGGCACCGGGTCAGG<br>CGCGGAAGCGAGCAGCCCAC<br>CGTCG
STRG.104813.1	*Halomicrobium*		none	Hv-YPC; GN101	> STRG.104813.1<br>GGACTCCAGTTTCAGGCCGT<br>GAAACCGCCGTTAGTGCGAT<br>GTAGCGCCGAAAACAATCAC<br>AACAATCACTAT
